# Retraction: MicroRNA-202 inhibits cell migration and invasion through targeting FGF2 and inactivating Wnt/β-catenin signaling in endometrial carcinoma

**DOI:** 10.1042/BSR-2019-0680_RET

**Published:** 2022-11-23

**Keywords:** cell invasion, cell migration, endometrial carcinoma, FGF2, miR-202

This article is being retracted from *Bioscience Reports* at the request of the authors as they have been unable to replicate the results found in this study, hence the authors feel that the conclusions drawn are inaccurate. The Editor-in-Chief and Editorial Board agree with the Retraction.

